# Tumor gene expression data classification via sample expansion-based deep learning

**DOI:** 10.18632/oncotarget.22762

**Published:** 2017-11-30

**Authors:** Jian Liu, Xuesong Wang, Yuhu Cheng, Lin Zhang

**Affiliations:** ^1^ School of Information and Control Engineering, China University of Mining and Technology, Xuzhou 221116, China

**Keywords:** gene expression data, classification, sample expansion, deep learning, 1-dimensional convolutional neural network

## Abstract

Since tumor is seriously harmful to human health, effective diagnosis measures are in urgent need for tumor therapy. Early detection of tumor is particularly important for better treatment of patients. A notable issue is how to effectively discriminate tumor samples from normal ones. Many classification methods, such as Support Vector Machines (SVMs), have been proposed for tumor classification. Recently, deep learning has achieved satisfactory performance in the classification task of many areas. However, the application of deep learning is rare in tumor classification due to insufficient training samples of gene expression data. In this paper, a Sample Expansion method is proposed to address the problem. Inspired by the idea of Denoising Autoencoder (DAE), a large number of samples are obtained by randomly cleaning partially corrupted input many times. The expanded samples can not only maintain the merits of corrupted data in DAE but also deal with the problem of insufficient training samples of gene expression data to a certain extent. Since Stacked Autoencoder (SAE) and Convolutional Neural Network (CNN) models show excellent performance in classification task, the applicability of SAE and 1-dimensional CNN (1DCNN) on gene expression data is analyzed. Finally, two deep learning models, Sample Expansion-Based SAE (SESAE) and Sample Expansion-Based 1DCNN (SE1DCNN), are designed to carry out tumor gene expression data classification by using the expanded samples. Experimental studies indicate that SESAE and SE1DCNN are very effective in tumor classification.

## INTRODUCTION

Tumors, which seriously endanger human health, are part of the major malignant diseases in the world. Early detection of the tumor is under a very important meaning for the better treatment of patients. The emergence and development of DNA microarray has promoted the research of tumor at the molecular level [1-3]. By mining the useful knowledge and information from the massive tumor gene expression data, we can have a comprehensive understanding of the nature of the tumor at the genetic level which plays an important role in promoting the clinical diagnosis and treatment of tumors as well as developing new drugs [4, 5]. Generally, gene expression data can be obtained from multiple tissue samples, including diseased samples and normal samples. By comparing the gene expression levels in diseased samples and normal samples, researchers can get a better insight into the disease pathology of the tumor [6, 7]. An urgent problem need to be addressed is how to effectively discriminate tumor samples from normal ones. To deal with this, many classification methods, such as Support Vector Machines (SVMs) [8] and Neural Networks [9]-[10], have been proposed for tumor gene expression data classification.

Among all classification methods, deep learning models show very good performance and draw more and more attention. Deep learning models have many advantages over conventional methods. On the one hand, deep learning models intrinsically learn a high level representation of the data so that avoiding laborious work [11]. On the other hand, deep structure has exponentially stronger expressive power than conventional shallow structure. Deep learning has achieved promising performance in many fields, such as computer vision, speech recognition, and natural language processing. According to [12], a review of deep learning in bioinformatics, deep learning models have also been widely used in the area of bioinformatics, including biomedical signal processing, biomedical imaging and omics. However, the application of deep learning is rare in tumor classification. The only available literature was written by Fakoor et al. [13]. Therefore, we attempt to use deep learning models to classify the tumor gene expression data.

Among a variety of deep learning models, Stacked Autoencoder (SAE) [14] is a widely used and effective method. SAE is a multi-layer neural network that reproduces the input signal as much as possible. It has been widely used in many areas, such as medical image processing [15], object recognition [16], and video classification [17]. In [13], SAE was successfully applied to the gene expression data for classifying the tumor samples. The classification process of SAE for a specific tumor type is given as follows: Firstly, the dimensionality of the feature space is reduced by using principle component analysis (PCA) due to the characteristics of the small sample problem in high-dimensional gene expression data. Secondly, other tumor gene expression data from the same platform are used as unlabeled data for feature learning since the number of samples for the specific tumor is really small. Thirdly, the weights of the features learned in the second step are tuned using the specific labeled data. Finally, the tumor gene expression data is classified. The drawback of literature [13] is that the gap between specific tumor data and other tumor gene expression data from the same platform is not considered, thus may have a negative effect on tumor classification. In this paper, we use SAE to achieve tumor classification in a different way with [13].

Convolutional Neural Network (CNN) [18] plays a dominant role in the community of deep learning models. CNN exploits spatially local correlation by enforcing a local connectivity pattern between neurons of adjacent layers. CNN has been demonstrated to provide better performance than other conventional methods on various vision tasks, such as face recognition [19], object detection [20], and image classification [21, 22]. In addition to vision tasks, CNN has also been applied to speech recognition [23, 24], natural language processing [25] and other fields. However, there are rare literatures on the technique with CNN for tumor classification. In this paper, CNN is considered to be applied to classify the tumor gene expression data.

CNN is the most widely used method in the field of image processing. Generally, a 2-dimensional image sample is taken as the input of CNN to implement convolution operation. However, each sample is a 1-dimensional array in tumor gene expression data which makes traditional CNN models not applicable for tumor classification. Fortunately, 1-dimensional CNN (1DCNN), a special CNN model, is proposed, and it requires the input is a 1-dimensional vector. 1DCNN has been used to analyze 1-dimensional sample in many areas. For example, Hu et al. have successfully utilized 1DCNN to process the spectral channels [26]. But the applicability of 1DCNN in the tumor gene expression data requires further study. Here, we introduced 1DCNN into tumor classification.

A large number of labeled data are usually required for training the deep learning models, including SAE and 1DCNN. However, the number of labeled samples of tumor gene expression data is quite small. For instance, there are 60 labeled samples in colon data [27] and only 20 labeled samples in breast cancer data [28]. In this paper, we propose a novel Sample Expansion (SE) method to address the problem of insufficient labeled samples. Inspired by Denoising Autoencoder (DAE) [29], a large number of labeled samples are obtained by randomly cleaning partially corrupted input many times. These labeled samples are taken as the expanded samples. Then we merged the expanded samples and untreated samples into a matrix as the training samples. This method can deal with the problem of insufficient training samples of tumor gene expression data to a certain extent. Furthermore, in order to benefit from both deep learning and SE, we suggest two deep learning-based methods, Sample Expansion-Based SAE (SESAE) and Sample Expansion-Based 1DCNN (SE1DCNN), for tumor classification. The tumor classification process is given as follows. Firstly, due to the high dimensionality of tumor gene expression data, we reduce the dimensionality of gene expression data. For each gene expression data, each feature represents one gene and has its natural meaning. Therefore, gene selection is more convincing than feature extraction in processing tumor gene expression data. Here, Infinite Feature Selection (Inf-FS) [30] is used as the dimensionality reduction strategy to select genes. Secondly, SE is implemented to expand the number of labeled samples. Finally, two deep learning models, SESAE and SE1DCNN, are utilized to achieve tumor classification based on the expanded samples. Experimental results demonstrate that SESAE and SE1DCNN are very effective in tumor gene expression data classification.

The main contributions of our work are summarized as follows. Firstly, for the first time, 1DCNN, an excellent deep learning model, is successfully applied to the tumor classification task. Secondly, a novel sample expansion method is proposed to deal with the problem of insufficient labeled samples when using deep learning models to implement tumor classification.

The remainder of the paper is structured as follows. In Section 2, SE is proposed and how to select cancer characteristic genes by SESAE or SE1DCNN is explained. Experimental results and discussion on tumor gene expression datasets are presented in Section 3. In Section 4, the conclusions are given.

## RESULTS AND DISCUSSION

This section shows the experimental results. In this paper, microarray data was used to perform our experiment. We performed our method on three publicly available gene expression datasets, i.e., breast cancer [28], leukemia [31] and colon cancer [27]. We determined the parameters of SESAE and SE1DCNN. To demonstrate the effectiveness of SESAE and SE1DCNN for tumor classification, 1DCNN [26], traditional SAE, SAE in [13], SAE with fine tuning in [13], and Softmax/SVM were employed for comparison. In this paper, the programs are implemented by using Python language and Theano library [32] on a PC equipped with an Intel Core i7 and Nvidia GeForce GTX 980 graphics card.

### Tumor gene expression datasets

We tested the proposed SESAE and SE1DCNN on three tumor datasets: breast cancer [28], leukemia [31] and colon cancer [27]. The statistics of the three datasets were summarized in Table 1. Inflammatory Breast Cancer (IBC) is a clinically defined variant of breast cancer characterized by its rapid onset and swollen, erythematous, and edematous presentation of the breast. The IBC dataset contains 30006 genes on 20 samples. There are two classes in 20 samples: 8 IBC samples and 12 non-IBC samples. Leukemia is a heterogeneous disease, usually caused by non-random chromosomal translocations that produce aberrant gene fusions or inappropriate expression of oncogenes and the prognosis for cure differs considerably among these genetic subtypes. Leukemia dataset contains 12600 genes on 60 samples. In [31], the 60 samples was processed into four classes: mercaptopurine alone (MP), high-dose methotrexate alone (HDMTX), high-dose methotrexate and mercaptopurine (HDMTX+MP), low-dose methotrexate and mercaptopurine (LDMTX+MP) and the corresponding number of samples are 12, 20, 10, 18. Colon cancer is a malignant tumor arising from the inner wall of the large intestine. In [27], colon cancer contains 2000 genes on 62 samples. There are 22 normal and 40 tumor colon samples.

**Table 1:** Summery of tumor gene expression datasets

Dataset	Data Labels	Number of	
		Genes	Samples
Breast cancer	1=non-IBC, 2=IBC	30006	20
Leukemia	1=MP, 2=HDMTX, 3=HDMTX+MP, 4=LDMTX+MP	12600	60
Colon cancer	1=cancer, 2= normal	2000	62

### Parameter determination

For each dataset, Inf-FS method was adopted as the dimensionality reduction algorithm to select genes. For fair comparison, 500 genes were selected by Inf-FS for each method. We performed 10-fold cross-validation and results were presented in terms of the average classification accuracy. In this subsection, the number of corrupted genes *a* was tested. For each *a*, we provided the parameters of SESAE and SE1DCNN on different tumor datasets. For SESAE and SE1DCNN, the choices of parameters might not be the best but effective for tumor classification.

Here, *a* = 1,2,3,4,5were tested. We tested the number of nodes of hidden layers in SESAE. Simultaneously, we also tested the number and size of convolution filters and the size of filters in max pooling. For each dataset, we took 20% samples of each class to expand the training samples and the rest 80% samples as testing samples. The parameters and classification accuracies of SESAE and SE1DCNN with different number of corrupted genes on breast cancer were summarized in Tables 2-3, respectively. In the case of *a* = 1,2,3,4,5, the number of training samples is 2505, 1255, 835, 630, 505, respectively. From Table 2, the best classification results of SESAE on breast cancer is 87.33% when *a* = 1. From Table 3, in the case of *a* = 1 and *a* = 2, SE1DCNN can reach the best performance 95.33%.

**Table 2:** The parameters and classification accuracies of SESAE with different number of corrupted genes on breast cancer

Layer		*a*=1	*a*=2	*a*=3	*a*=4	*a*=5
Hidden Layer1	Number of Nodes	50	50	50	50	50
Hidden Layer2	Number of Nodes	50	50	50	50	50
Accuracy (%)		87.33	86.67	86.00	86.67	86.00

**Table 3:** The parameters and classification accuracies of SE1DCNN with different number of corrupted genes on breast cancer

Layer		*a*=1	*a*=2	*a*=3	*a*=4	*a*=5
C1 Filter	Number	11	11	5	11	11
	Size	21	21	21	21	21
M1 Filter	Size	4	4	4	4	4
C2 Filter	Number	5	5	5	5	5
	Size	21	21	21	21	21
M2 Filter	Size	4	4	4	4	4
Accuracy (%)		95.33	95.33	93.33	94.67	94.00

The parameters and classification accuracies of SESAE and SE1DCNN with different number of corrupted genes on leukemia dataset were summarized in Tables 4-5, respectively. When *a* = 1,2,3,4,5, the number of training samples is 6513, 3263, 2171, 1638, 1313, respectively. From Table 4, the best classification result of SESAE is 49.79% when *a* = 1. From Table 5, the best performance of SE1DCNN is 57.87% when *a* = 1.

**Table 4:** The parameters and classification accuracies of SESAE with different number of corrupted genes on leukemia dataset

Layer		*a*=1	*a*=2	*a*=3	*a*=4	*a*=5
Hidden Layer 1	Number of Nodes	30	30	30	30	30
Hidden Layer 2	Number of Nodes	30	30	30	30	30
Accuracy (%)		49.79	49.36	48.72	48.30	48.51

**Table 5:** The parameters and classification accuracies of SE1DCNN with different number of corrupted genes on leukemia dataset

Layer		*a*=1	*a*=2	*a*=3	*a*=4	*a*=5
C1 Filter	Number	22	17	22	9	17
	Size	21	21	21	21	21
M1 Filter	Size	4	4	4	4	4
C2 Filter	Number	5	5	5	16	5
	Size	21	21	21	21	21
M2 Filter	Size	4	4	4	4	4
Accuracy (%)		57.87	57.02	57.24	56.17	55.96

The parameters and classification accuracies of SESAE and SE1DCNN with different number of corrupted genes on colon cancer were summarized in Tables 6-7, respectively. When *a* = 1,2,3,4,5, the number of training samples is 6513, 3263, 2171, 1638, 1313, respectively. From Table 6, the best classification result of SESAE is 84.49% when *a* = 1. From Table 7, the best performance of SE1DCNN is 85.51% when *a* = 2.

**Table 6:** The parameters and classification accuracies of SESAE with different number of corrupted genes on colon cancer

Layer		*a*=1	*a*=2	*a*=3	*a*=4	*a*=5
Hidden Layer 1	Number of Nodes	100	100	100	100	100
Hidden Layer 2	Number of Nodes	100	100	100	100	100
Accuracy (%)		84.49	83.68	83.28	83.89	83.69

**Table 7:** The parameters and classification accuracies of SE1DCNN with different number of corrupted genes on colon cancer

Layer		*a*=1	*a*=2	*a*=3	*a*=4	*a*=5
C1 Filter	Number	25	5	20	12	20
	Size	21	21	21	21	21
M1 Filter	Size	4	4	4	4	4
C2 Filter	Number	20	10	7	9	5
	Size	21	21	21	21	21
M2 Filter	Size	4	4	4	4	4
Accuracy (%)		84.90	85.51	85.30	84.49	85.10

Furthermore, we expanded all the samples, and tested whether the corrupted samples can be correctly classified. The classification accuracies of SESAE and SE1DCNN on three datasets with different *a* were provided in Table 8. For breast cancer, in the case of *a* = 1, SESAE and SE1DCNN have the best results 99.88% and 99.94%, respectively. For leukemia, in the case of *a* = 1, SESAE and SE1DCNN have the best results 99.78% and 99.84%, respectively. For colon dataset, in the case of *a* = 1, SESAE and SE1DCNN have the highest accuracies 99.96% and 99.98%, respectively. The results indicate that the corrupted samples can be correctly classified and the meaningful features are successfully captured by the corrupted samples.

**Table 8:** The classification accuracies (%) of SESAE and SE1DCNN on three datasets with different values of *a* when all the samples are expanded

Dataset	Method	*a*=1	*a*=2	*a*=3	*a*=4	*a*=5
Breast Cancer	SESAE	99.88	99.78	99.75	99.53	99.49
	SE1DCNN	99.94	99.84	99.81	99.74	99.68
Leukemia	SESAE	99.78	99.55	99.46	99.20	98.94
	SE1DCNN	99.84	99.67	99.54	99.37	99.12
Colon Cancer	SESAE	99.96	99.94	99.93	99.86	99.86
	SE1DCNN	99.98	99.95	99.93	99.88	99.86

### Comparison with other methods

To demonstrate the effectiveness of SESAE and SE1DCNN for tumor classification, traditional 1DCNN [26], traditional SAE, SAE in [13], SAE with fine tuning in [13], and Softmax/SVM were employed for comparison. SAE in [13] and SAE with fine tuning in [13] use other tumor gene expression data from the same platform to achieve feature learning since the number of labeled samples in tumor data is really small. The results were shown in Table 9. The best performance in Table 9 was indicated by bold.

**Table 9:** The classification accuracies (%) of different methods on three datasets

Methods	Breast Cancer	Leukemia	Colon Cancer
SE1DCNN	**95.33**	**57.87**	**84.90**
1DCNN	86.00	51.49	83.67
SESAE	87.33	49.79	84.49
SAE	80.67	32.55	82.07
SAE in [13]	63.33	33.71	66.67
SAE (Fine tuning) in [13]	83.33	33.71	83.33
Softmax/SVM	85.0	46.33	83.33

On all the three datasets, SE1DCNN has better performance than all the other methods. On colon and breast cancer datasets, except for SE1DCNN, SEASE outperforms the other methods. On leukemia dataset, except for SE1DCNN and 1DCNN, SESAE have the best performance among all the 5 methods. This indicates that our SE method is very effective in classifying tumor data. Without SE method, 1DCNN outperforms traditional SAE, SAE in [13], SAE with fine tuning in [13], and Softmax/SVM on breast and colon cancer. Except for SE1DCNN, 1DCNN has better performance than the other methods, including SESAE, on leukemia dataset. The performance of SE1DCNN and 1DCNN demonstrate that 1DCNN is a powerful method when achieving tumor classification.

## METHODOLOGY

### Sample expansion method

**Figure 1:**
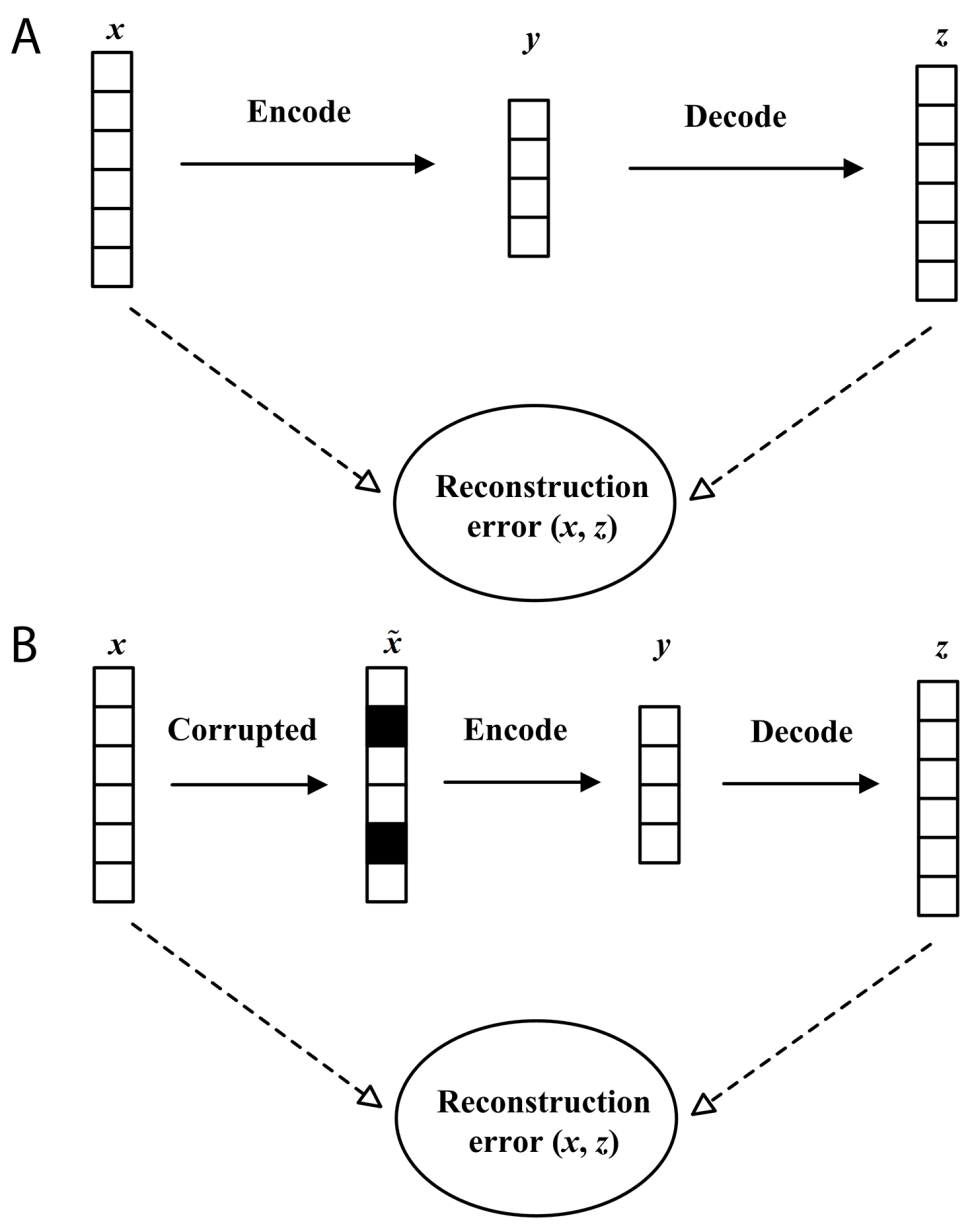
The graphical representation of Autoencoder **(A)** and Denoising Autoencoder **(B)**.

In the classification problem, it is particularly critical to obtain a good feature representation. The traditional Autoencoder (AE) can learn a useful representation by encoder. However, we cannot obtain robust features by using Autoencoder. A very different strategy is proposed by Vincent et al. to get a high-level representation: cleaning partially corrupted input, or in short denoising [29]. There are two underlying ideas in this strategy: Firstly, a good representation should be robust and stable when the input is damaged; Secondly, denoising is required to extract features that obtain useful structure in the input distribution. This denoising strategy was successfully used into Autoencoder and Denoising Autoencoder. The graphical representation of AE and DAE is described in Figure 1. In Figure 1(A), denote a vector ***x*** as an input firstly. Secondly, ***x*** is mapped to ***y*** via an encoder. Thirdly, the Autoencoder attempts to reconstruct ***x*** by decoding, ***y*** and generates the reconstruction vector ***z***. Finally, the Autoencoder calculates the reconstruction error between ***x*** and ***z***. In Figure 1(B), DAE performs some different operations compared with Autoencoders. Firstly, raw data ***x*** is stochastically corrupted to $\tilde{x}$. In $\tilde{x}$, each value filled with black is forced to be 0. Secondly, the corrupted data $\tilde{x}$ is mapped to ***y*** via an encoder. Thirdly, DAE reconstructs ***x*** by decoding ***y***, and generates the reconstruction vector ***z***. Finally, DAE calculates the reconstruction error between ***x*** and ***z*** with a loss function.

The process of denoising, that is, mapping a corrupted sample back to an uncorrupted one, can be given an intuitive geometric interpretation under the so-called manifold assumption, which states that natural high dimensional data concentrates close to a non-linear low-dimensional manifold. Based on uncorrupted samples ***X***, corrupted samples $\tilde{X}$ obtained by applying corruption process $q\left(\tilde{X}|X\right)$. During denoising training, we learn a stochastic operator $p\left(\tilde{X}|X\right)$ that maps a corrupted $\tilde{X}$ back to its uncorrupted ***X***, Corrupted samples are much more likely to be outside and farther from the manifold than the uncorrupted ones. Thus stochastic operator $p\left(\tilde{X}|X\right)$ learns a map that tends to go from lower probability points $\tilde{X}$ to nearby high probability points ***X***, on or near the manifold. Note that when $\tilde{X}$ is farther from the manifold, $p\left(\tilde{X}|X\right)$ should learn to make bigger steps, to reach the manifold. Successful denoising implies that the operator maps even far away points to a small region close to the manifold. The denoising idea can thus be seen as a way to define and learn a manifold. And it can better learn a higher level representation which is rather stable and robust under corruptions of the input. The detailed interpretation of denoising idea can be founded in [29]. As a kind of natural high dimensional data, gene expression data also has the manifold structure. So the denoising idea can be used to analyze gene expression data. Experiments in [29] show that the corrupted data is very useful. There are two main reasons can explain this result: Firstly, the corrupted data can be trained to obtain smaller weight noise than non-corrupted data; Secondly, the corrupted data reduces the generation gap between the training and testing data to a certain extent [29].

Thanks to the denoising idea, in this paper, a novel Sample Expansion method is proposed to address the problem of insufficient training samples for tumor gene expression data. Denote $X\in {\mathbb{R}}^{m\times n}$ as a tumor dataset with *m* genes and *n* samples. For each sample in ***X***, SE method randomly chooses a(a≤m) genes and corrupts corresponding values to 0. Supposing the locations of the corrupted genes are non-repeated, we repeat this process floor(m/a) times, where *floor*() is a function that is rounded down, and it guarantee the number of expanded samples is an integer. And each processed sample is saved for future operations. In this approach, floor(m/a) expanded samples can be obtained from one sample. Similarly, n×floor(m/a) expanded samples can be obtained from all *n* samples. Finally, n×floor(m/a) expanded samples and *n* raw samples are merged into one matrix to be taken as training data.

**Figure 2:**
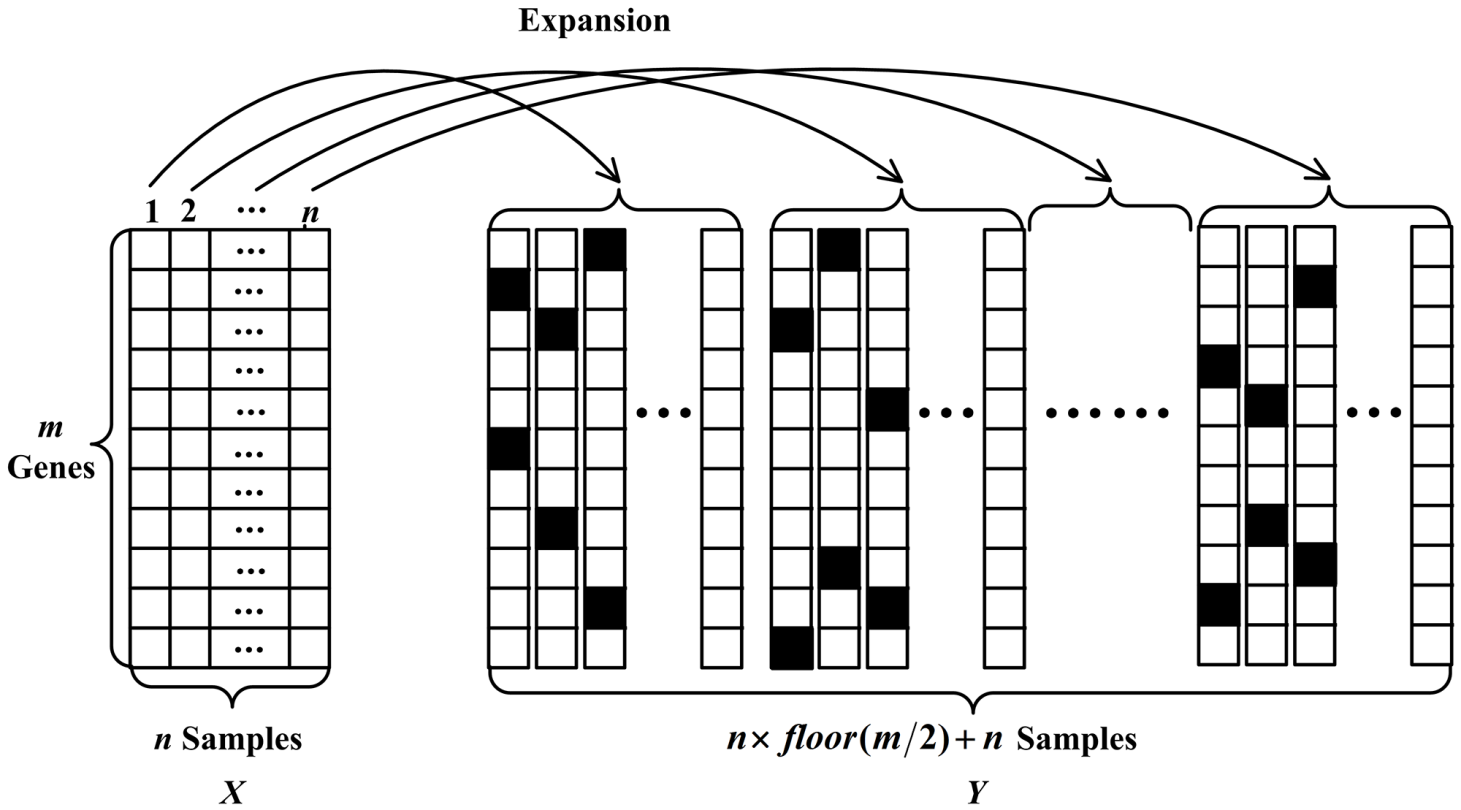
The schematic representation of sample expansion method

We give the visualization of SE method in Figure 2. Denote a tumor gene expression dataset as $X\in {\mathbb{R}}^{m\times n}$ with each row representing a gene and each column representing a sample. To be more specific, here, we set *a* to 2. In Figure 2, the values of the corrupted genes are filled with black in expanded samples. Statistically, for the first sample in ***X***, floor(m/2) expanded samples are obtained by using SE method. Including the first sample in ***X***, floor(m/2)+1 samples are obtained from one sample. Other samples in ***X*** are processed in the same manner. Finally, n×floor(m/2)+n samples are stored in a matrix ***Y*** that can be taken as the training data. By utilizing SE, a large number of training samples can be obtained. The expanded samples maintain the merits of the corrupted data and address the problem of insufficient training samples of gene expression data to a certain extent.

We attempt to interpret the rationality of SE from a biological perspective. Generally, the differential expression of multiple genes may be expected to result in various diseases. Moreover, the realization of a biological process usually requires interaction of multiple genes. Unfortunately, we cannot accurately determine which combinations of genes are the decisive ones we want. After being processed by SE, the non-corrupted genes in each expanded sample are taken as a combination. The corrupted samples can generate a variety of gene combinations. Some of these gene combinations may indicate distinct biological processes or different gene co-expression [33]. From this perspective, the class of samples can be represented more effectively by large number of gene combinations, thereby improving the classification accuracy.

### Sample expansion-based SAE

An Autoencoder usually has three layers: one input layer, one hidden layer, and one reconstruction layer (See Figure 1(A)).

During training, the input ***x*** is mapped to the hidden layer and produces the latent activity ***y***. This step is called an encoder and can be formulated as follows

$$y=f\left(W_{y}x+b_{y}\right),$$ (1)

where ***W***_***y***_ denotes the input-to-hidden weights, ***b***_***y***_ denotes the bias of hidden units, and *f* (·) denotes the activation function. Here, the sigmoid function is taken as the activation function.

Then, ***y*** is mapped to a reconstruction layer by a decoder. The reconstructed value is denoted as ***z***. This step can be written as

$$z=f\left(W_{z}x+b_{z}\right),$$ (2)

where ***W***_***z***_ denotes the hidden-to-output weights, ***b***_***z***_ denotes the bias of output units.

In this paper, we hold the following constraint: ***W***_***y***_ =***W***_***z***_ =***W*** This can help to halve model parameters. Therefore, three groups of parameters, ***W***, ***b***_***y***_ and ***b***_***z***_, need to be learned.

The goal of Autoencoder is to minimize the reconstruction error between ***x*** and ***z***

$$\underset{W,b_{y},b_{z}}{\text{arg}\;\text{min}}\;\;\text{cost}\left(x+z\right),$$ (3)

where *cost* (***x, z***) denotes the reconstruction error. The weight updating rule can be defined as

$$W=W-\eta \frac{\partial \text{cost}\left(x\mathrm{,z}\right)}{\partial W},$$ (4)

$$b_{y}=b_{y}-\eta \frac{\partial \text{cost}\left(x\mathrm{,z}\right)}{\partial b_{y}},$$ (5)

$$b_{z}=b_{z}-\eta \frac{\partial \text{cost}\left(x\mathrm{,z}\right)}{\partial b_{z}},$$ (6)

where *η* denotes the learning rate.

After the model training, the learned feature lies in the hidden layer, which can be used for classification. It can also be used as the input of a higher layer to learn a deeper feature in deep learning models. The power of Autoencoder lies in the form of reconstruction-oriented training. During reconstruction, Autoencoder only uses the information in ***y***. If an Autoencoder perfectly recovers the input from ***y***, ***y*** can maintain enough information of the input. In addition, the learned nonlinear transformation in ***y*** can be regarded as a good feature extraction process. Therefore, stacking the encoders can minimize the loss of information in data. In the meantime, the abstractive and invariant information can be preserved in the deeper features. All these characteristics promote us to choose Autoencoder to extract deep features for tumor gene expression data.

**Figure 3:**
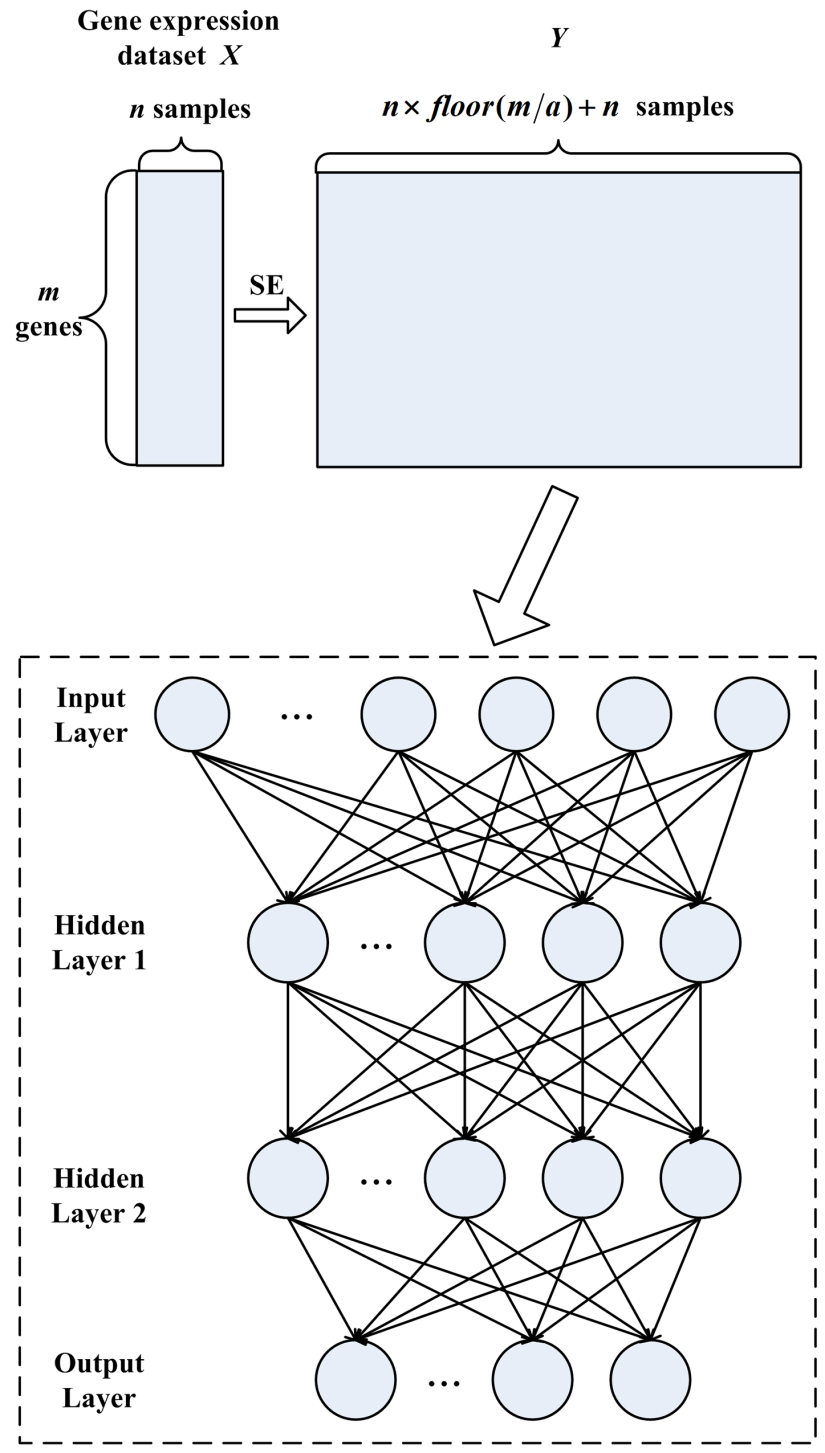
The SESAE architecture consisting of sample expansion process, one input layer, two hidden layers and one output layer

The Stacked Autoencoder (SAE) can be constructed by stacking the input and hidden layers of Autoencoder together. The SESAE is designed by applying the SE method to SAE for tumor classification. The SESAE architecture is given in Figure 3. It consists of one input layer, two hidden layers and one output layer. SESAE first implements SE method to obtain a large number of labeled samples. Then the expanded samples and raw samples are fed into SAE. SAE first maps inputs in Input Layer to Hidden Layer 1. This step is similar with Autoencoder. After the training of Hidden Layer 1 in SAE, the inputs of subsequent layer of SAE are the output of the previous layer. We try to reconstruct the output of Hidden Layer 1according to the activity of Hidden Layer 2. After this, the decoder of the Hidden Layer 2 is cast away, and only the input-to-hidden parameters are incorporated as weights between Hidden Layer 1 and Hidden Layer 2. The subsequent classifier is also implemented as a neural network. We adopt fine-tuning strategy to adjust the parameters during training procedure. Here, we train the classifier using the back propagation method that searching for a minimum in a peripheral region of parameters initialized by the former step.

### Sample expansion-based 1DCNN

CNN is a classical deep learning model that exploits spatially local correlation by enforcing a local connectivity pattern between neurons of adjacent layers. CNN architecture consists of various combinations of convolutional layers, max pooling layers and fully-connected layers. Neurons in the same convolutional layer are sparsely connected to the neurons in the next layer and share the same weight. Weight sharing can reduce the number of trainable parameters and make CNN an effective model. The output of a convolutional layer is usually taken as the input of a max pooling layer. Max pooling layers divide the input into multiple non-overlapping windows and output the maximum value for each window. Max pooling can reduce the computation complexity for upper convolutional layers and provide translation invariance of features from the location. In the classification task, a fully-connected layer is used to integrate all the feature maps of the last pooling layer.

The training process of CNN contains two key steps: forward propagation and back propagation. The former step computes the actual classification results with current parameters while the later step updates the trainable parameters to narrow the gap between the actual classification results and the desired classification results.

Denote ***x***^*i*^ as the output of the *i*-th layer and the input of the next layer. Define ***x***^*i*^ to be

$$x^{i}=f(u^{i}),$$ (7)

with

$$u^{i}=W^{i}\;x^{i-1}+b^{i},$$ (8)

where ***W***^*i*^ is a weight matrix of the *i* -th layer and ***b***^*i*^ is an additive bias vector of the *i* -th layer. In Eq. (7), *f* (*u*^*i*^) is the activation function of the *i* -th layer. In this paper, the Rectified Linear Unit (RELU) is taken as the activation function. For a classification problem with *C* classes and *N* training samples, the squared-error loss function [34] is given as

$$J^{N}=\frac{1}{2}\sum_{n=1}^{N}\sum_{c=1}^{C}{\left(t_{c}^{n}-y_{c}^{n}\right)}^{2},$$ (9)

where $t_{c}^{n}$ is the *c*-th class of the *n*-th label, $y_{c}^{n}$ is the value of the *c*-th output layer unit in response to the *n*-th label.

Due to the error over the whole dataset is a sum over the individual errors on each sample, the backpropagation is considered with respect to a single sample. The error function of the *n*-th sample is

$$J^{n}=\frac{1}{2}\sum_{c=1}^{C}{\left(t_{c}^{n}-y_{c}^{n}\right)}^{2}.$$ (10)

The errors can be regarded as sensitivities of each unit with respect to perturbations of ***b***, that is

$$\frac{\partial J}{\partial b}=\frac{\partial J}{\partial u}\frac{\partial u}{\partial b}.$$ (11)

Since ∂u/∂b=1, we can define

$$\delta =\frac{\partial J}{\partial u}.$$ (12)

This derivative plays a decisive role in the back propagation from higher layers to lower layers. The following formula is used to implement the back propagation

$$\delta^{i}={\left(W^{i+1}\;\right)}^{Τ}\delta^{i+1}\circ f^{\prime }(u^{i}),$$ (13)

where ∘ is element-wise multiplication. The sensitivities for the output layer neurons take a different form

$$\delta^{\mathrm{output}}=f^{\prime }\left(u^{i}\right)\circ (y^{n}-t^{n}).$$ (14)

Finally, the delta rule is used to update weights and biases for the neurons. For the *i* -th layer, the weight is updated by

$$\frac{\partial J}{\partial W^{i}}=\frac{\partial J}{\partial u^{i}}\frac{\partial u^{i}}{\partial W^{i}}={\left(\delta^{i}\right)}^{T}x^{i-1},$$ (15)

$$\Delta W^{i}=-\eta \frac{\partial J}{\partial W^{i}},$$ (16)

where *η* is the learning rate. The bias is updated by

$$\frac{\partial J}{\partial b^{i}}=-\eta \frac{\partial J}{\partial u^{i}}\frac{\partial u^{i}}{\partial b^{i}}=\delta^{i},$$ (17)

$$\Delta b^{i}=-\eta \frac{\partial J}{\partial b^{i}}.$$ (18)

With the increase of the number of iterations, the value of the loss function is smaller which indicates the actual output is closer to the desired output. Finally, CNN can be utilized to classify the dataset.

**Figure 4:**
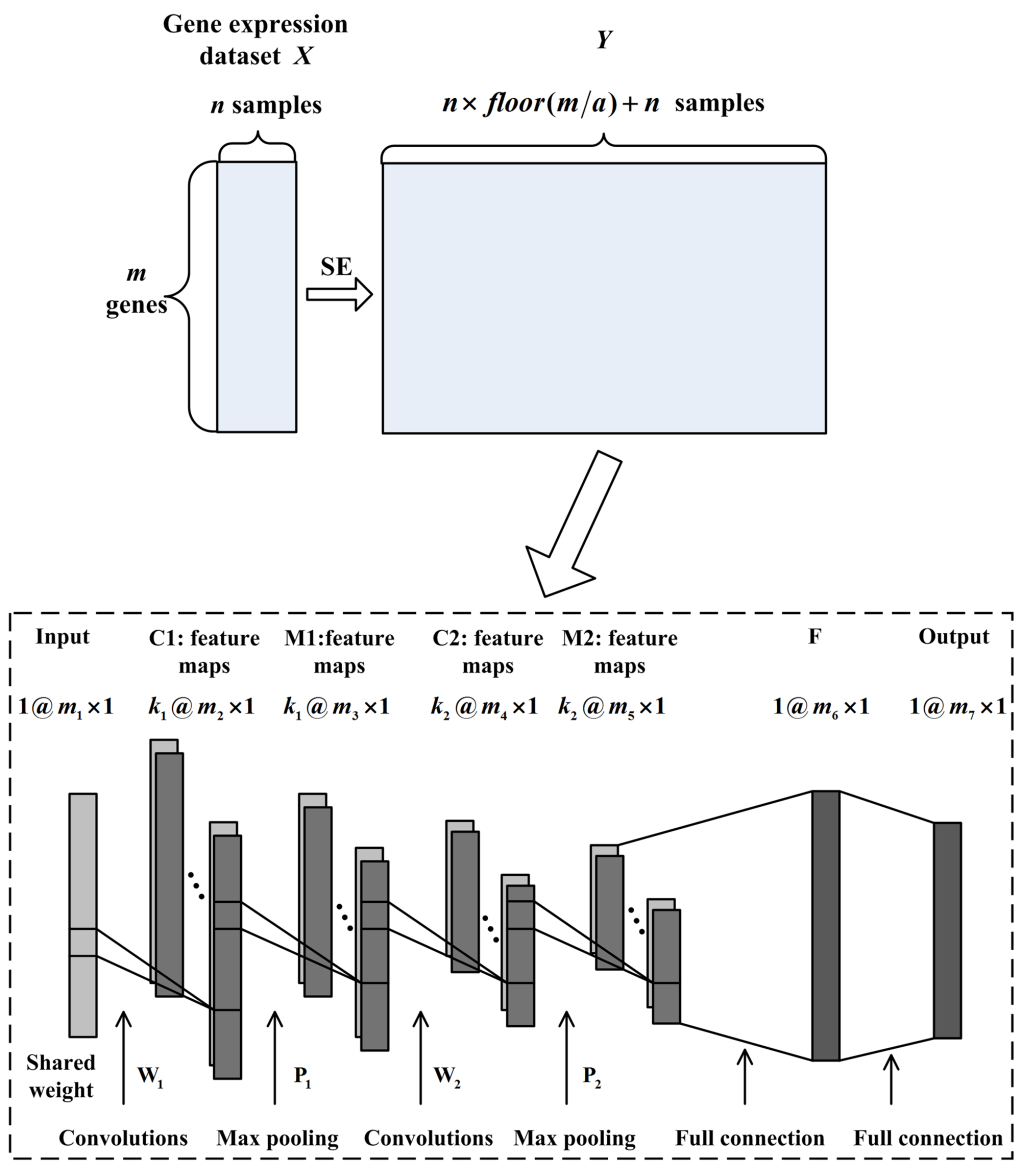
The SE1DCNN architecture consisting of sample expansion process, two convolutional layers, two max pooling layers and one fully-connected layer

In image processing, the input of CNN should be a 2-dimensional image for convolution. However, each sample of gene expression data is a 1-dimensional array and the above process cannot be achieved. Therefore, 1DCNN, which asks for a 1-dimensional vector as input, is introduced in this paper. By combining the SE method and 1DCNN, we design the SE1DCNN to implement tumor classification task. In Figure 4, the designed architecture of SE1DCNN is shown. Except for the sample expansion process, 1DCNN has 7 layers: one input layer, two convolutional layers C1 and C2, two max pooling layers M1 and M2, one fully-connected layer F and one output layer. In each tumor gene expression dataset, each sample can be taken as the input of 1DCNN. In Figure 4, the input layer is a sample with *m*_1_ genes. Suppose **W**_1_ with size *w*_1_ × 1 and **W**_2_ with size *w*_2_ × 1 are the convolutional kernels of the first and second convolutional layers C1 and C2, respectively; **P**_1_ with size *p*_1_ × 1 and **P**_2_ with size *p*_2_ × 1 are the filtering kernels of the first and second max pooling layer M1 and M2, respectively; *k*_1_ and *k*_2_ are the number of kernels. After convolving the input layer, C1 contains *k*_1_ × *m*_2_ × 1 nodes where *m*_2_ = *m*_1_−*w*_1_ + 1. M1 contains *k*_1_ × *m*_3_ × 1 nodes where $m_{3}=m_{2}/p_{1}$. C2 contains *k*_2_ × *m*_4_ × 1 nodes where *m*_4_ = *m*_3_−*w*_2_ + 1. M2 contains *k*_2_ × *m*_5_ × 1 nodes where $m_{5}=m_{4}/p_{2}$. The fully-connected layer F and the output layer contain *m*_6_ and *m*_7_ nodes, respectively.

The classification process of SE1DCNN is the same as that of CNN. The main difference between SE1DCNN and CNN is that SE1DCNN has a sample expansion step and needs a 1-dimensional vector as input.

### Tumor classification via sample expansion-based deep learning

In tumor classification task, the management of high-dimensional gene expression data requires an efficient feature selection method to individuate redundant and/or irrelevant features and avoid overfitting [35]. In [30], Roffo et al. proposed a novel unsupervised feature selection method dubbed Infinite Feature Selection (Inf-FS). The feature selection problem is mapped to an undirected fully-connected graph without label information in Inf-FS. Then a subset of features is considered as a path to connect vertices in the graph. The cost of the path, which is embedded into a cost matrix, is implemented by the combination of pairwise relationships between features and is modeled as a function of both standard deviation and Spearman’s rank correlation coefficient. By construction, Inf-FS allows exploiting the convergence properties of power series of matrices, and the relevance and redundancy of one feature with respect to all the other features are calculated.

Inf-FS is an excellent feature selection method by ranking the importance individuates candidate features. The most appealing characteristic of Inf-FS is that the importance of a feature is assessed by considering all the possible subsets of all the features. In addition, the relevance and redundancy of each feature are influenced by all the other ones. Numerous experiments demonstrate that Inf-FS outperforms many classical feature selection methods, such as SVM-RFE [8], Fisher [36] and Relief-F [37]. Therefore, we adopt Inf-FS as the dimensionality reduction strategy to select genes from gene expression data.

**Figure 5:**
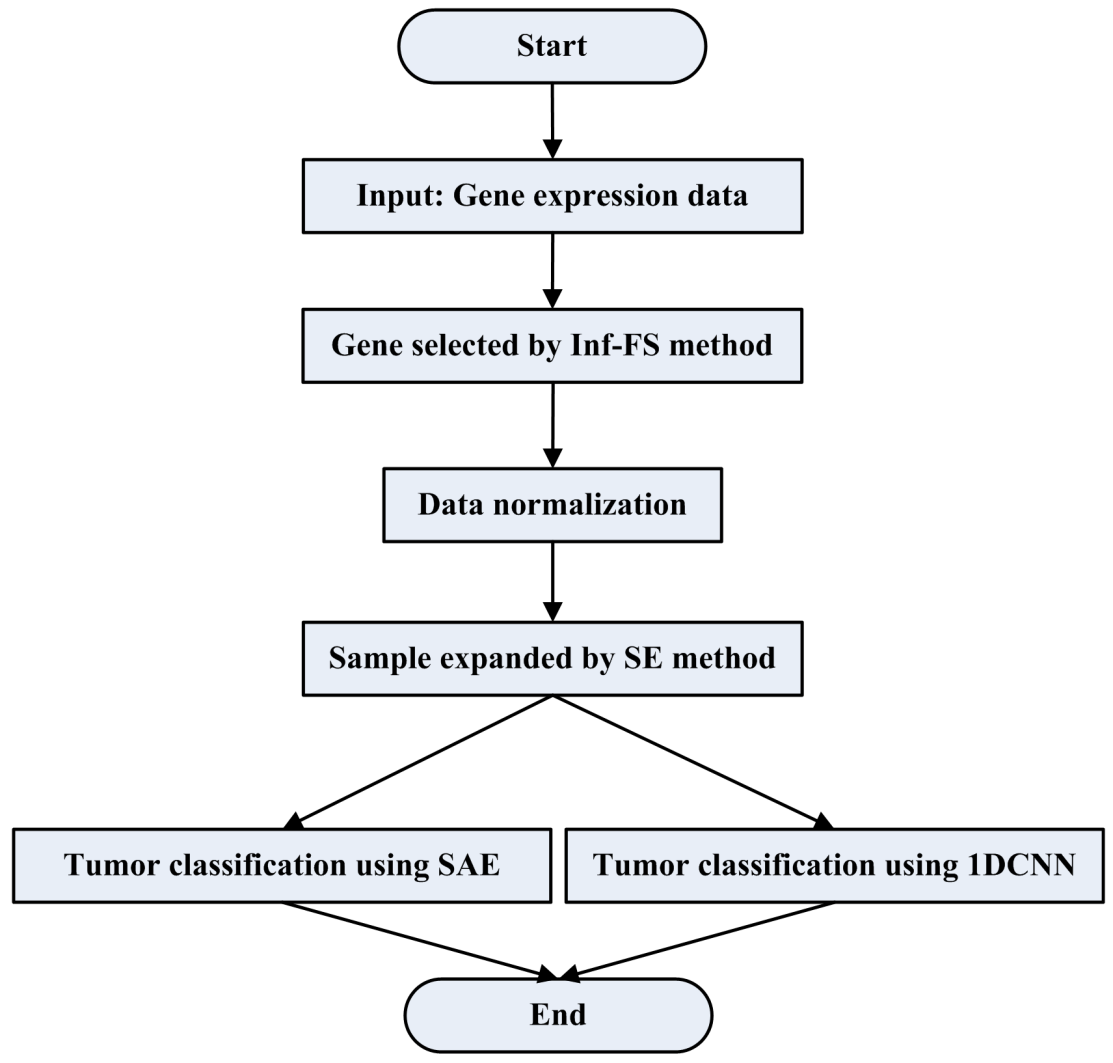
The flowchart of tumor classification by using SESAE or SE1DCNN

In this paper, the framework of tumor gene expression data classification via Sample Expansion-based deep learning is given in Figure 5.

Firstly, Inf-FS is used as the dimensionality reduction strategy to select genes.

Secondly, the dimensionality reduced data is normalized. We use the following normalization

$$\hat{X}=\left(X-\mathrm{mean}\left(X\right)\right)\frac{\mathrm{std}\left(\hat{X}\right)}{\mathrm{std}\left(X\right)}+\mathrm{mean}\left(\hat{X}\right),$$ (19)

where *mean*(***X***) is the mean of the dimensionality reduced data matrix ***X*** by row, *std*(***X***) is the standard deviation of ***X*** by row, $\mathrm{std}\left(\hat{X}\right)$ is the standard deviation of the expected matrix $\hat{X}$ by row and $\mathrm{mean}\left(\hat{X}\right)$ is the mean of the expected matrix $\hat{X}$ by row. Here, $\mathrm{std}\left(\hat{X}\right)$ and $\mathrm{mean}\left(\hat{X}\right)$ are simply set to 1 and 0 respectively.

Thirdly, SE method is used to obtain a large number of training samples. The gene expression data is divided into two parts: training data and testing data. We use SE method to increase the number of labeled training data by using the separated training data.

Finally, two deep learning models, SAE and 1DCNN, are adopted to classify tumor data. The expanded and raw training data are merged into a matrix that is used as the new training data to train SAE and 1DCNN. We test SESAE and SE1DCNN by using the testing data.

## CONCLUSIONS

In this paper, two sample expansion-based deep learning models, Sample Expansion-Based Stacked Autoencoder (SESAE) and Sample Expansion-Based 1D Convolutional Neural Network (SE1DCNN), are designed to classify tumor gene expression data. Firstly, since feature selection is more believable than feature extraction, an excellent feature selection method Inf-FS is used to reduce the dimensionality of gene expression data. Secondly, inspired by the denoising idea in DAE, a Sample Expansion method is proposed. The expanded samples not only have the benefits of corrupted data in DAE but also solve the problem of insufficient labeled training samples of gene expression data to a certain extent when using deep learning models. We also give an interpretation of SE method from a biological perspective. Finally, since SAE and CNN can provide excellent classification effect in many fields, the applicability of SAE and 1DCNN on gene expression data is discussed. A 4-layer SAE and a 7-layer 1DCNN are designed to classify the tumor gene expression data by using the expanded samples and raw samples.

We tested the proposed SESAE and SE1DCNN on three tumor datasets: breast cancer, leukemia and colon cancer. We first provided the parameters of SESAE and SE1DCNN on different tumor datasets with different ^*a*^. This is a guide to the choice of parameters. Moreover, we expanded all samples to determine the effectiveness of the corrupted samples. The high classification accuracies of SESAE and SE1DCNN on three datasets demonstrate that the corrupted samples are very useful. Traditional 1DCNN, traditional SAE, SAE in [13], SAE with fine tuning in [13], and Softmax/SVM are employed for comparison. The classification results indicate that SE1DCNN has the best performance than the competitive methods on all the three datasets. Except for SE1DCNN and 1DCNN, SEASE outperforms the other methods on all three datasets. The performance of SESAE and SE1DCNN suggests that joint SE method and deep learning models can effectively achieve tumor gene expression data classification. The main reason is that SE method provides more and useful training samples for two deep learning models. In addition, we also find that except for SE1DCNN, 1DCNN has better performance than the other methods on leukemia dataset. And except for SE1DCNN and SESAE, 1DCNN outperforms the other methods on breast cancer and colon cancer datasets. Experimental studies on SE1DCNN and 1DCNN indicate that 1DCNN is more efficient than the other methods for tumor classification.

The limitation of this paper is mainly the explanation of SE method from a biological perspective is insufficient. In this paper, we give a short interpretation of SE method from a biological perspective. We believe that the non-corrupted genes in expanded samples can be taken as a gene combination and the corrupted samples can generate a variety of gene combinations. These gene combinations may indicate distinct biological processes or different gene co-expression. From this perspective, the class of samples can be represented more effectively by large number of gene combinations, thereby improving the performance of tumor classification. The pathogenesis of the tumor needs to be studied in future to discover the useful combinations of genes. By analyzing these combinations of genes, our SE method may give a more persuasive interpretation. In future, we will focus on the biological meaning of different combinations of genes to propose a more reasonable sample expansion strategy for tumor classification when using deep learning.
